# Scalability and scaling-up strategy of a physical activity policy intervention in Australian childcare centres

**DOI:** 10.1093/heapro/daaf145

**Published:** 2025-09-03

**Authors:** Hayley Christian, Matthew Mclaughlin, Andrea Nathan, Emma Adams, Adrian Bauman, Patti-Jean Naylor, Trevor Shilton, Carol Maher, Stewart G Trost, Jasper Schipperijn

**Affiliations:** The Kids Research Institute Australia, The University of Western Australia, 15 Hospital Avenue, Nedlands, Perth, WA 6009, Australia; School of Population and Global Health, The University of Western Australia, 35 Stirling Highway, Crawley, Perth, WA 6009, Australia; The Kids Research Institute Australia, The University of Western Australia, 15 Hospital Avenue, Nedlands, Perth, WA 6009, Australia; School of Medicine and Public Health, College of Health, Medicine and Wellbeing, University of Newcastle, University Drive, Callaghan, NSW 2038, Australia; The Kids Research Institute Australia, The University of Western Australia, 15 Hospital Avenue, Nedlands, Perth, WA 6009, Australia; School of Population and Global Health, The University of Western Australia, 35 Stirling Highway, Crawley, Perth, WA 6009, Australia; The Kids Research Institute Australia, The University of Western Australia, 15 Hospital Avenue, Nedlands, Perth, WA 6009, Australia; School of Population and Global Health, The University of Western Australia, 35 Stirling Highway, Crawley, Perth, WA 6009, Australia; School of Public Health, University of Sydney, A27 Fisher Road, Sydney, NSW 2050, Australia; Department of Sports Science and Clinical Biomechanics, University of Southern Denmark, Camusvej 55, Odense 5230, Denmark; School of Exercise Science, Physical and Health Education, University of Victoria, PO Box 1700 STN CSC, Victoria, BC, Canada V8W 2Y2; School of Public Health, Curtin University, GPO Box U1987, Bentley, WA 6845, Australia; Alliance for Research in Exercise, Nutrition and Activity (ARENA) Allied Health and Human Performance, City East Campus, University of South Australia, GPO Box 2471, Adelaide, SA 5001, Australia; School of Human Movement and Nutrition Sciences, The University of Queensland, Level 2, Connell Building, St Lucia, Brisbane, QLD 4072, Australia; Department of Sports Science and Clinical Biomechanics, University of Southern Denmark, Camusvej 55, Odense 5230, Denmark

**Keywords:** scalability, scale-up, physical activity, childcare, intervention, policy

## Abstract

There is an urgent need for scalable interventions to promote physical activity in early childhood. An early childhood education and care (ECEC) physical activity policy intervention with implementation support strategies (Play Active) has been proposed for scale-up in Australia. This study sought to assess the scalability of Play Active and describe the Play Active scaling-up strategy. The Intervention Scalability Assessment Tool was used to assess scalability. The PRACTical planning for Implementation and Scale-up (PRACTIS) guided the scaling-up strategy and involved: (i) characterizing the implementation setting; (ii) identifying existing/new partnerships; (iii) identifying barriers and facilitators to implementation; (iv) addressing barriers through adaptations. The Play Active scalability assessment domains with the highest scores (>2.5/3) were for the problem, intervention, reach and acceptability. Four additional domains scored highly (>2/3): fidelity and adaptation, delivery settings and workforce, implementation infrastructure, and strategic/political context. The lowest scores (<2/3) were the evidence of effectiveness, intervention costs and benefits, and sustainability domains. The PRACTIS guide showed that the implementation setting and existing and new partnerships were appropriate for scaling-up Play Active. The PRACTIS guide also identified key barriers (e.g. staff time) and enablers (e.g. staff professional development) to implementation at scale. Adaptations were identified to address these barriers (e.g. intervention delivery via a customised website). Overall, the scalability assessment revealed gaps in some scalability domains to be addressed through further research and adaptation of Play Active. The proposed scale-up trial evaluation is crucial to support decision-makers to fund, scale and institutionalize Play Active in the real world.

Contribution to Health Promotion

- Building on scant empirical guidance, this paper outlines the scalability of, and the scaling-up strategy to support the implementation of physical activity interventions in early childhood education and care.
- Stakeholder involvement is key to both the scalability assessment and the development of a theory-informed scaling-up strategy.
- Concurrently describing a scalability assessment of a proposed physical activity policy intervention (Play Active) and a theory-informed scaling-up strategy provides an example of best practice in scale-up.
- The systematic approach taken to develop the Play Active scaling-up strategy can be applied to other interventions and settings.

## INTRODUCTION

Physical activity has compelling benefits from childhood through to adulthood (Jones *et al*. 2013, Carson *et al*. 2017). During the early years, it improves children’s wellbeing, learning, physical and mental health, and social skills (Carson *et al*. 2017). However, a large proportion of young children do not achieve the 1 h of energetic play per day required as part of the recommended 3 h of total daily physical activity (Tapia-Serrano *et al*. 2022, Christian *et al*. 2024). There is a clear need to intervene at scale to increase physical activity during early childhood.

The World Health Organization has identified early childhood education and care (ECEC) as a key setting for promoting children’s physical activity (World Health Organization 2021). However, a systematic review of physical activity interventions in ECEC identified just two studies reporting on scaled-up ECEC interventions, defined as more than 50 ECEC services receiving the intervention (Wolfenden *et al*. 2020). Since this review, there have been two evaluations of ECEC physical activity interventions delivered at scale (Hassani *et al*. 2020, Kracht *et al*. 2020, Tugault-Lafleur *et al*. 2022), including the rollout of a provincial government mandatory policy (Active Play Standards) and accompanying capacity building initiative (Appetite to Play) in British Columbia, Canada (Hassani *et al*. 2020). Appetite to Play was found to be scalable and positively impacted ECEC provider knowledge, confidence and intentions (Hassani *et al*. 2020), highlighting the promise of scaling-up interventions for improving children’s physical activity in ECEC.

While many tools exist to guide implementation and scale-up [e.g. PRACTical planning for Implementation and Scale-up (PRACTIS) guide (Koorts *et al*. 2018); Exploration, Preparation, Implementation, Sustainment framework (Aarons *et al*. 2011); Stages of Implementation Completion (Chamberlain *et al*. 2011)] they are typically underused in the planning stages of scaling-up physical activity interventions (O'Hara *et al*. 2014, Cassar *et al*. 2019, Moullin *et al*. 2019). Ideally, implementation efforts should begin with dissemination and sustainment in mind using implementation and scale-up tools throughout a project (O'Hara *et al*. 2014, Moullin *et al*. 2019, McKay *et al*. 2024).

Systematic reviews suggest physical activity interventions can remain effective once scaled but typically lose approximately half of their effect (McCrabb *et al*. 2019, Lane *et al*. 2021, Sutherland *et al*. 2022). This ‘scale-up penalty’ may in part be overcome by addressing two parallel challenges: ‘if’ an intervention should be scaled up (scalability assessment) and ‘how’ best to scale it up (the scaling-up strategy). Determining ‘if’ an intervention should be scaled up requires assessment across several scalability domains such as the proposed intervention’s cost, political acceptability, and effectiveness (Milat *et al*. 2020), using tools such as the Intervention Scalability Assessment Tool (ISAT) (Lee *et al*. 2020, Milat *et al*. 2020). To maintain intervention effects at scale, adaptations should have a clear goal, be guided by evidence-informed frameworks such as the Model for Adaptation Design and Impact (MADI) (Kirk *et al*. 2020), and be fidelity consistent (Kirk *et al*. 2020).

To date, there has been a lack of attention given to scaling-up strategies (i.e. ‘how’ to scale), a gap addressed by the PRACTIS guide, which provides four key steps for characterizing implementation settings, engaging stakeholders, and identifying and addressing implementation barriers (Koorts *et al*. 2018). It is important the scaling-up strategy is done in partnership with stakeholders and that the scaled-up intervention is a priority for those stakeholders. Thus, the aim of this study was to assess the scalability of a physical activity policy intervention for ECEC (Play Active) using the ISAT and describe the Play Active scaling-up strategy using the PRACTIS guide. The research questions were: (i) Is the Play Active intervention for ECEC scalable for delivery in other Australian states and territories? and (ii) What is the scaling-up strategy for delivering the Play Active intervention at scale?

## METHODS

### The play active policy intervention (pre-scale-up)

The setting for this pragmatic trial was long-day care services for children 5 years and under. Most participating services were privately owned, consistent with the broader Australian ECEC landscape. The central component of Play Active is an evidence-informed physical activity policy template for ECEC (Christian *et al*. 2020, Nathan *et al*. 2022). The Play Active policy provides nine age-specific recommendations on the amount of physical activity and sedentary time (including screen time) children should have whilst at ECEC and 25 physical activity practices that ECEC services should implement (Christian *et al*. 2020). Six implementation support strategies were developed to support ECEC services implement their Play Active policy: (i) personalized policy for each service; (ii) policy review and approval; (iii) a resource guide; (iv) a brief assessment tool for monitoring children’s energetic play; (v) professional development/training; and (vi) Project Officer support (Nathan *et al*. 2022). Play Active was evaluated through a pragmatic cluster randomized trial in 2021–2022 (*n* = 81 ECEC services; 3-to-5-month Play Active policy implementation period) (Nathan *et al*. 2022). Play Active resulted in significantly higher uptake of physical activity policy practices (*P* = 0.034), had high awareness among educators (90%), and was deemed acceptable (83%) with high fidelity and reach of implementation support strategies (>75%) (Adams *et al*. 2023).

### Scalability assessment

Scalability of the Play Active intervention was assessed using the ISAT (Lee *et al*. 2020, Milat *et al*. 2020). Part A of the ISAT focuses on setting the scene (domains A1–A5) and Part B focuses on intervention implementation planning (domains B1–B5). It includes 50 guiding questions across 10 domains, informing 19 scored questions (response range zero to three). The 10 domains are: (A1) the problem; (A2) the intervention; (A3) strategic/political context; (A4) evidence of effectiveness; (A5) intervention costs and benefits; (B1) fidelity and adaptation; (B2) reach and acceptability; (B3) delivery setting and workforce; (B4) implementation infrastructure; and (B5) sustainability.

The scalability assessment procedure involved two steps. First, data and information were mapped to each of the 50 guiding questions across the 10 domains. Multiple data sources were triangulated including published findings (Adams *et al*. 2023, Wenden 2023) and input from a Consumer Advisory Group (comprised of 12 parents) and a Partner Advisory Group (comprising partners from ECEC, government, play and public health sectors). Second, four research team members (H.C., M.M., A.N., and E.A.) and three representatives from the Partner Advisory Group (Cancer Council Western Australia; Goodstart Early Learning; Western Australian Department of Local Government, Sport and Cultural Industries) involved in both the pragmatic cluster randomized trial (Nathan *et al*. 2022) and the scaling-up strategy independently scored Play Active on the 19 ISAT questions. All scorers had a university degree or higher and 57% were female. Each person was provided with an overview of the ISAT as well as the ISAT tool with the data/information mapped to each domain. Ethics approval was obtained from The University of Western Australia Human Research Ethics Committee (RA/4/20/6120 and 2023/ET000187).

Mean scores for each domain were calculated, resulting in 10 domain scores ranging from zero (least scalable) to three (most scalable). Mean scores were plotted on a radar plot. The mean scores are descriptive and relative to one another, allowing comparison across the ten scalability domains. The results were reported in line with guidance for reporting the ISAT Summary Assessment (Lee *et al*. 2020). Based on the ISAT results, the overall suitability of the intervention for scale-up was determined (i.e. whether it merits scale-up; it is promising, but further information/planning is warranted; or it does not merit scale-up).

### Scaling-up strategy development

The initial phase of the scaling-up strategy (Simmons and Shiffman 2007) involved engaging key partners involved in the pragmatic cluster randomized trial (Nathan *et al*. 2022) and other stakeholders and funders who were interested in scaling-up Play Active to multiple Australian states and territories. The Play Active scaling-up strategy was guided by the four-step PRACTIS guide (Koorts *et al*. 2018). The parameters of the implementation setting (Step 1) were summarized using the recommended PRACTIS guide 5 P’s; place, people, process, provision and principles (Koorts *et al*. 2018). Step 2 involved engaging with key stakeholders via the established Play Active Partner Advisory Group. For Step 3, the barriers and facilitators to implementation were identified through qualitative interviews and focus groups with ECEC directors and educators (Wenden 2023), process evaluation data from the pre-scale-up pragmatic cluster randomized trial (Adams *et al*. 2023), and the literature (Wolfenden *et al*. 2015, Finch *et al*. 2016, Move Munch and Move Healthy Eating Program 2021). In Step 4, the MADI tree (Kirk *et al*. 2020) guided decisions about proposed adaptations to the existing (pre-scale-up) Play Active implementation support strategies to address potential barriers (identified in Step 3) to effective implementation at scale. Full details of the methods and findings of Step 4 have been published in detail elsewhere (McLaughlin *et al*. 2023). Finally, each Play Active implementation strategy proposed for scale-up was categorized according to the Expert Recommendations for Implementing Change (ERIC) (Waltz *et al*. 2015).

## RESULTS

### Scalability assessment

**Figure 1.**
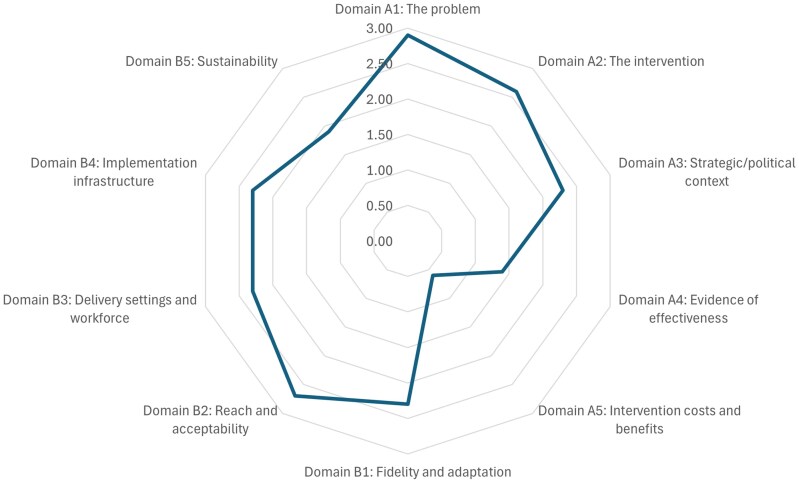
Radar plot of the ISAT scores for Play Active.

Figure 1 shows the radar plot for the Play Active ISAT scores for each domain. The highest scores (>2.5/3) were for the problem, intervention, and reach and acceptability domains. The lowest scores (<2/3) were the evidence of effectiveness, intervention costs and benefits, and sustainability domains. Overall, the results identified that the Play Active intervention merited scaling-up. The mean scores and justification for each are summarized below (the full details are included in Supplementary Table S1).

#### Domain A1: the problem (2.9/3)

Despite the importance of physical activity for young children’s health and development and ECEC being a key setting to promote young children’s physical activity, prior to Play Active, there was no ECEC-specific guidance on the amount of physical activity and sedentary time (including screen time) children should have whilst attending ECEC in Australia. Furthermore, just 16% of ECEC services in Western Australia were found to mention children’s physical activity in their service policies (Christian *et al*. 2020). Thus, Play Active was deemed to be addressing a current issue.

#### Domain A2: the intervention (2.6/3)

As described in the methods, the evidence-informed Play Active policy (i.e. the intervention) contains 25 practices focused on the: manager, supervisor and educator (*n* = 14); physical environment (*n* = 4); family and carer (*n* = 5); and policy monitoring and review (*n* = 2). The Play Active policy is accompanied by six implementation support strategies. The score was lower as contextual barriers in ECEC, such as sector-wide workforce challenges (United Workers Union 2021), are not able to be addressed by the intervention.

#### Domain A3: the strategic and political context (2.3/3)

The Play Active intervention is aligned with the Australian National Quality Standards for ECEC Quality Area 2.1: ‘Each child’s health and physical activity is supported and promoted’ (Australian Children's Education and Care Quality Authority 2023). However, there is currently no mandate for ECEC services to have a physical activity policy.

#### Domain A4: the evidence of effectiveness (1.4/3)

Play Active was evaluated in a pragmatic cluster randomized controlled trial within 81 ECEC services in Perth, Western Australia, which demonstrated significant increases in the uptake of Play Active policy practices. Despite increased practices, high acceptability and good programme fidelity, there was no significant change in children’s physical activity, likely related to the short implementation timeframe for a new policy.

#### Domain A5: intervention costs and benefits (0.6/3)

To the authors’ knowledge, there are no published trials reporting the cost-effectiveness of physical activity interventions in ECEC, and as such, this may be a barrier to adoption and implementation (Asada *et al*. 2023). Little is known about the cost of the Play Active intervention from the initial trial, as an economic evaluation was not possible.

#### Domain B1: fidelity and adaptation (2.3/3)

The planned adaptations to the implementation support strategies, including their extent, type, fidelity consistency, goals, size, scope and anticipated impact, are summarized here with further detail available. Briefly, a total of 53 adaptations were identified for the delivery of Play Active in different Australian states (Western Australia, South Australia and Queensland). The main goals for adapting Play Active were to increase the acceptability, appropriateness, and feasibility, decrease costs, and increase adoption of the intervention. Most adaptations (68%) were made to the content of the implementation strategies. For example, providing online access to resources via a dedicated Play Active website, rather than printed materials alone. More than half (56%) of the adaptations involved adding elements for scale-up (e.g. tailored prompts to complete professional development). The delivery mode of implementation support strategies also changed from phone call and email support to website-based delivery. Most adaptations were fidelity consistent (95%). Adaptations were primarily small-to-medium in size, with most perceived to have a positive (87%) or neutral (8%) effect on the effectiveness of the intervention, rather than negative (4%) (McLaughlin *et al*. 2023).

#### Domain B2: reach and acceptability (2.7/3)

Given there are more than 100 000 children attending ECEC across almost 3000 ECEC services in Western Australia, South Australia and Queensland, the Play Active intervention has large potential reach (Department of Education 2024). The Play Active intervention was found to be highly acceptable in the pre-scale-up trial, with 83% of educators and 78% of directors reporting it as acceptable. Play Active is likely to be acceptable at scale, as it retains the key features (e.g. a physical activity policy, professional development, and resources) of the pre-scale-up trial.

#### Domain B3: delivery settings and workforce (2.3/3)

The delivery setting is ECEC services across Western Australia, South Australia and Queensland. This is broader than the initial evidence-generating trial, which focused on the Perth metropolitan area, Western Australia. The implementation workforce will be a project officer in each state and an overall project manager who will deliver the implementation support strategies with the support of a customized automated website. There is a relatively smaller workforce needed for the proposed scale-up of Play Active compared to the initial trial, due to increased efficiencies of the automated website delivery.

#### Domain B4: implementation infrastructure (2.3/3)

The infrastructure requirements to implement Play Active were designed to be feasible for scale-up. The multi-modal delivery of implementation support includes a custom-built website, physical resource package (i.e. membership pack) and a community of practice. The use of drop-shipping and print-on-demand services will reduce the need for implementation infrastructure for storage and postage. However, the score is influenced by the fact that implementation staff are not already embedded in the workforce.

#### Domain B5: sustainability (1.9/3)

As the Play Active intervention is partly research-funded there will be a nominal membership fee (approximately AUD200) to cover the cost of receiving the physical resources in the membership pack. For scale-up and sustainability, there is a deliberate focus on website automation to reduce workforce requirements. However, website maintenance, adaptations, and regular updates will be required. Overall, little is known about the sustainability of the Play Active intervention, as the initial trial had a seven-to-nine-month follow-up period. A key objective of scaling-up Play Active is to determine its long-term sustainability and potential for roll out to other Australian states and territories.

### Scalingup strategy

#### Characterizing the parameters of the implementation setting (step 1)

The parameters (place, people, process, provision, principles) of the implementation setting are summarized in Table 1.

**Table 1.** The Five P’s describing the parameters of the implementation setting (place, people, process, provision, principles)

Criteria	Description	Summary
People	The type and number of people that the intervention will reach, and the individuals that will be involved/required for implementation and scale-up.	Long day-care ECEC services across Western Australia (*n* = 776), South Australia (*n* = 445) and Queensland (*n* = 1744) catering for approximately 100 000 children (Finch *et al*. 2016).
Process	The intervention or implementation process that will occur in practice	Through a purpose-built website, ECEC service directors can become a Play Active member by completing a self-assessment of 25 questions, eight brief online professional development videos, a policy tailoring process and membership. This process is expected to take 60 min. Through the same purpose-built website, educators complete the same eight brief professional development videos and receive a certificate of completion. This process is expected to take 20 min and will be mobile-phone friendly. Educators and directors will also receive ongoing prompts via email and SMS, access to resources and a newsletter, for the full 2-year membership period. Services are able to renew their membership after 2 years.
Provision	The resources (e.g. human, physical and fiscal) that will be necessary to achieve intervention implementation and scale-up	Website: Registration, self-assessment, brief professional development, policy tailoring, membership, online access to resources and member benefits (e.g. discount codes to further professional development opportunities), resource guide. Human resources: The Kids Institute Research Institute will employ Project Officers in each state, led by a Project Manager, to deliver the implementation support strategies and policy, on behalf of the Partner Advisory Group. Mail: Resource pack for services who are Play Active members (including large sign displaying Play Active logo, as well as printed resources such as posters).
Principles	The underlying principles of the intervention (e.g. individual behaviour change) and implementation process (e.g. building capacity for implementation) that will be scaled-up in practice	Intervention targets individual and environmental change based on socio-ecological theory. Implementation—purpose-built website to facilitate automation of processes and minimize human resources (e.g. certification process is automated).

#### Identifying and engaging key stakeholders (step 2)

Play Active was co-produced with the Play Active Partner Advisory Group. They have met formally (quarterly) and informally since 2019 and are involved in the scaling-up of Play Active. The Play Active Partner Advisory Group is comprised of representatives from different sectors and different states, as recommended by the PRACTIS guide. Representatives include Goodstart Early Learning; Early Childhood Australia; Cancer Council Western Australia; YMCA Western Australia; Play Matters Collective; Western Australian Department of Local Government Sport and Cultural Industries; Health and Wellbeing Queensland; Australian Childcare Alliance Western Australia; Australian Childcare Alliance Queensland; Australian Childcare Alliance South Australia; Play Australia; Nature Play Australia; Western Australian Department of Health; Sonas Early Learning; Sagewood Early Learning; and the National Heart Foundation.

##### Intervention funding/responsibility

The Kids Research Institute Australia is responsible for delivering the intervention, acting on behalf of the Partner Advisory Group and Chief Investigators. These groups have secured research funding to support the next phase, which includes the delivery and evaluation of the intervention in a scale-up trial (2023–27). In addition, ongoing discussions are taking place with partners to explore options for the long-term, sustainable delivery of the Play Active programme beyond the scope of the current research.

##### Intervention dissemination

The dissemination/recruitment strategy was co-developed with the Play Active Partner Advisory Group. All members of the advisory group have a role to distribute the intervention to promote recruitment. A key strategy for recruitment was including ECEC service providers (e.g. Goodstart Early Learning) in the Partner Advisory Group to increase ownership through co-designing the intervention, which in turn will support dissemination and recruitment.

##### Intervention host/implementation

The project team based at The Kids Research Institute Australia will work with the Play Active Partner Advisory Group to deliver the Play Active intervention.

##### Intervention users

The primary target users are ECEC service directors and educators. A secondary target user is parents of children attending ECEC.

#### Identifying implementation barriers and facilitators (step 3)

Barriers and facilitators were identified using process evaluation data from the pre-scale-up pragmatic cluster randomized trial, post-trial qualitative data, stakeholder input and the existing literature. Barriers and facilitators were identified across all PRACTIS guide levels: individual-level (e.g. knowledge and skills of educators), provider-level (e.g. leadership/support from management), organizational-level (e.g. resources and funding availability), and community/systems-level (e.g. parent engagement and low-paid workforce with lack of time for professional development). A summary of the key barriers and facilitators is in Table 2.

**Table 2.** Summary of the barriers and facilitators to promoting children’s physical activity in ECEC services^a^

	Barriers	Facilitators
Individual-level (educators)	**Knowledge and skills of educators**—lack of training and/or professional development relating to physical activity	**Professional development** *—*children’s physical activity topics (building children’s physical activity knowledge and skills; relationships between physical activity and healthy eating; management of risk and physical activity; restructuring environments/physical spaces; the ‘how and why’ of a physical activity policy); continual professional development, rather than one-off; shared learnings and continuous improvement; recognition/accreditation
	**Educator perceptions**—physical activity can cause/worsen injury or illness (safety mindset, reinforced by parental beliefs); children do not want to be active; weather constraints (too hot, too cold, too wet)	**Educator practices** *—*modelling of physical activity practices (for children, other staff); physical activity champion; planning and programming for children’s physical activity
	**Educator attitudes*—***negativity towards physical activity; reluctance to change established routines	**Educator attitudes—**More confidence in supporting and prioritizing children’s physical activity
	**Existing workloads and competing priorities**—Prioritizing children’s academic readiness (reinforced by parental perceptions/messages)	**Adequate resourcing*—***equipment, funding, space (reallocating so more space for physical activity), staff, time
	**Parent engagement** *—*difficulty engaging with parents about physical activity	**Supportive policy** *—*service has and implements a physical activity policy; included as part of new staff and family induction; discussed at staff meetings
Provider-level (directors)	**Inadequate leadership/support from management**	**Strong leadership/support by management**
	**Lack of educator support/buy in**	**Champions—**educators who are physical activity champions
	**Competing priorities*—***Director not interested in physical activity	**Assessment*—***self-assess children’s levels of physical activity in service; have and implement an evidence-based physical activity policy in service
Organizational-level (ECEC service/provider and broader)	**Cost and resourcing—**insufficient staffing; funding not available; lack of time (to do professional development); insufficient equipment (fixed and portable play equipment); lack of space available (indoors and outdoors); inadequate resourcing (computers to access information, training and resources)	**Resources and funding are available—**mobile friendly professional development; equitable access and culturally appropriate resources and training
	**Competing priorities—**too many policies and procedures to implement	**Children’s physical activity is a priority—**children’s physical activity is a strategic priority and planned for; discussed at staff team meetings; improving educator physical activity practices is a key focus; parent engagement around children’s physical activity is prioritized
	**Changes in leadership/management**	**Supportive policy—**physical activity policies exist and are evidence-based, clear and concise; standards and legislation in place to promote children; s physical activity
		**Supportive physical environments*—***access to green space nearby ECEC services; sufficient space indoors and outdoors, variety of fixed and portable play equipment
Community-level (parents)	**Parent perceptions and attitudes—**child is already very active; injuries/illness from being physically active; not a learning priority (school readiness more important); lack of interest/priority in physical activity; conflicts with other behaviours (e.g. screen time)	**Supportive educator-parent relationship—**educators communicate with parents re child’s physical activity behaviour at ECEC, benefits of physical activity, programmes and policies have in place, what the home physical activity behaviour and environment is like; use different forms of communication; communication is clear and consistent and evidence-based
		**Parents provided with resources and information**—on supporting children’s physical activity; resources are culturally appropriate and there is equitable access
		**Parent-parent champions*—***promotion of physical activity programmes and policies implemented in their child’s ECEC service

^a^Barriers and facilitators were identified using process evaluation data from the pre-scale-up pragmatic cluster randomized trial, post-trial qualitative data, stakeholder input, and the existing literature.

#### Addressing potential barriers to implementation (with adaptations) (step 4)

The resulting adaptations have been described in detail and previously published. The ten implementation support strategies are presented in Table 3, mapped to the barriers they address (Step 3), and ERIC strategies (Waltz *et al*. 2015).

**Table 3.** Play active implementation support strategies and associated ERIC implementation categories and barriers addressed

Implementation support strategy	ERIC strategy alignment	Barriers addressed	Implementation support sub-strategies
Professional development	Identify and prepare champions Make training dynamic	Skills and expertise of educators Perception physical activity can cause/worsen injury or illness Difficulty engaging with parents about physical activity Educator negativity towards physical activity (attitudes and/or practices) Perceptions that children do not want to be active Educator perceptions of weather constraints (e.g. too hot, too cold, too wet)	Play Active professional development videos (8–10) Delivered via Play Active website Educators and directors complete Can be completed via mobile Short (60–90 s each) Address barriers and facilitators Short quiz to assess knowledge gained Personalized completion certificate
Tailoring of physical activity policy	Mandate change Promote adaptability	Lack of leadership/support from service Directors Too many policies to implement	Results from self-assessment survey (completed by Directors) used to tailor service’s Play Active policy Self-assessment survey includes 25-items about the service’s current physical activity practices Seven high impact low effort policy practices pre-selected Hard and e-copy of Play Active policy
Tailored prompts and positive reinforcement	Distribute educational materials Remind clinicians (ECEC directors and educators) Tailor strategies	Lack of support/buy-in from educators Too many policies to implement	Automated prompts (email/SMS) sent for actions (e.g. registering with Play Active) and in-action (e.g. to complete professional development) Tailored to individuals, specific to website stage progressing through to become a member
Survey and feedback reports	Audit and provide feedback Develop and implement tools for quality monitoring Purposely re-examine the implementation	Lack of leadership/support from service directors	Director self-assessment survey to measure service’s current physical activity practices. Completed at baseline, 12 and 24 months after become a member Educator self-assessment survey to measure amount of physical activity and sedentary time provided to children. Completed at baseline, 12 and 24 months after become a member At 12 and 24 months summary progress report provided to services
Membership including resources	Change accreditation or membership requirements Create or change credentialing and/or licensure standards Develop educational materials	Lack of support/buy-in from educators Costs in time, resources, and money Leadership changes Skills and expertise of educators Perception physical activity can cause/worsen injury or illness Difficulty engaging with parents about physical activity Educator negativity towards physical activity (attitudes and/or practices) Perceptions that children do not want to be active Educator perceptions of weather constraints (e.g. too hot, too cold, too wet)	Membership pack for each service (including tailored Play Active Member certificate, Play Active policy, Resource Guide, Posters, Sign, Stickers, Parent flyers) Play Active website members area dashboard with resources Play Active Resource Guide is mapped to the 25 policy practices Monthly e-alerts sent specific to their tailored Play Active policy If individuals change services their account details can be updated
Community of practice	Capture and share local knowledge Centralize technical assistance Create a learning collaborative	Lack of support/buy-in from ECEC staff	Quarterly e-newsletter Fortnightly e-alerts Testimonials and sharing of Play Active experiences through website and socials Presentations—online and in person Live webinars
Parent Resources	Develop educational materials Involve patients/consumers and family in feedback	Parental perception of their child’s physical activity levels is high Physical activity injury/illness concerns Lack of time for/interest in physical activity Don’t perceive physical activity as a learning opportunity Parental expectations of the ECEC service for school readiness Conflicts with rules and norms at home (e.g. around screen time)	Parent section on Play Active website with resources and information Play Active social media Quarterly e-newsletter Co-developed with Parent Consumer Reference Group
Membership Renewal Process	Conduct ongoing training	Lack of support/buy-in from ECEC staff	Membership renewal reminders (automated via website) Membership renewal to include review and update of service’s Play Active policy Access to new resources and training via the Play Active website members dashboard
Branding and Partnerships	Build a coalition Develop academic partnerships Develop resource sharing agreements Obtain formal commitments Use advisory board and workgroups	Lack of support/buy-in from ECEC staff	Brand guidelines Communication plan Stakeholder/partner engagement plan Partner logo lock up on Play Active website and resources In partnership with Partner Advisory Group
Increase demand (Dissemination/recruitment strategy)	Increase demand Use mass media	Lack of support/buy-in from ECEC staff	Play Active Dissemination strategy/marketing campaign including a paid social media

## DISCUSSION

This paper presents the results of a scalability assessment and describes the scaling-up strategy for a proposed scaled-up version of the ECEC-based Play Active physical activity policy intervention with accompanying implementation support strategies. The scalability assessment domains with the highest scores were for the problem, intervention, and reach and acceptability. Four additional domains scored highly, including fidelity and adaptation, delivery settings and workforce, implementation infrastructure, and strategic/political context. The lowest scores suggested the least evidence existed for effectiveness, intervention costs and benefits, and sustainability domains. To the authors’ knowledge, there is only one other detailed example of the prospective use of the PRACTIS guide to inform the scaling-up strategy of a physical activity intervention. A study by McGrath *et al*. (2022) used the PRACTIS guide to steer the implementation of a ‘men’s shed’ intervention in Ireland. The authors suggested that the scaling-up strategy (i.e. how to scale up) was as important as the outcome of the scaled-up intervention, emphasizing the importance of investing time in partner relationships and the meaningful involvement of partners in the scaling-up strategy. For Play Active, the proposed implementation support strategies prioritize partner involvement and co-design, emphasizing that ongoing partnerships are an important strategy for the Play Active intervention.

The version of the Play Active intervention proposed for scale-up was awarded research funding which provides an opportunity to continue to improve the Play Active intervention and implementation support strategies in relation to sustainability, effectiveness and cost-effectiveness. This next phase of the research includes an economic evaluation to determine the cost-effectiveness of the Play Active scaled-up intervention. The scaled-up version of Play Active will also have a focus on programme sustainability whereby ECEC services will be encouraged to renew their Play Active membership after 2 years. There are also proposed strategies to secure the longer-term delivery of the programme by government and/or non-government partners and for the research team to stay engaged in the programme’s ongoing evaluation. Further evidence of the effectiveness of Play Active when delivered at scale will be collected by measuring change in ECEC services policy practices and educator-reported levels of children’s physical activity over a longer follow-up period. A sub-study will capture children’s physical activity levels at ECEC using device-based measures. Finally, the newly funded study is designed to support ECEC services to implement their Play Active policy over a longer period than the pre-scale-up trial (i.e. a minimum 12-month compared with 3-month period at pre-scale-up). It is expected that further adaptations to Play Active will be required, and these will be recorded and assessed to determine the effect on implementation and children’s physical activity in ECEC. Tools such as the PRACTIS guide (10) and MADI (24) will be used to guide this process.

### Strengths and limitations

A key strength of this study was the use of the ISAT to conduct a scalability assessment and the use of the PRACTIS guide to develop the scaling-up strategy for Play Active. This included input from the Play Active Partner Advisory Group and Consumer Advisory Group, a review of the barriers and facilitators to implementation, and theory-informed adaptations using the MADI framework. To the authors’ knowledge, this is the first prospective application of the PRACTIS guide to inform a scaling-up strategy for a physical activity intervention. Future research should consider developing a scaling-up strategy to help plan for the scaling-up of physical activity interventions. A further strength was the involvement of partners in the scalability assessment, the identification of, and the addressing of lower scoring scalability domains in the next phase of the research. However, future research should consider the number and characteristics of ISAT scorers including socio-demographic factors, and child physical activity background as well as their level of engagement in the prior research and the planned scaled-up intervention. Given that funding has now been secured to deliver the scaled-up version of Play Active statewide across Western Australia, South Australia and Queensland, it will be possible to evaluate the scaling-up strategy and scalability of the intervention in an ongoing way, as further adaptations and modifications are required. A limitation of the research is that the scaling-up strategy, including the proposed adaptations, is specific to the Australian ECEC context and may not be generalizable to the ECEC setting in other countries. However, the systematic approach taken to developing the Play Active scaling-up strategy can be applied to other interventions and settings. Implementing an equity lens to understand how scaled-up interventions perform in diverse settings, including in regional and remote communities will also require further investigation (McLoughlin and Salmon 2024).

## CONCLUSION

The proposed Play Active implementation support strategies were deemed suitable for scale-up, based on a scalability assessment. However, the scalability assessment revealed gaps and challenges in certain scalability domains that may be addressed through future research and adaptation. If the scaling-up strategy is effective and there is an improvement in ECEC educators’ physical activity-related practices and children’s physical activity levels it will provide the necessary evidence to support decision-makers to fund, scale and deliver Play Active in the real world. By documenting Play Active’s scaling-up strategy prior to scale-up and then evaluating its effectiveness, the findings can be used to inform future scale-ups. It will also support future comparison studies that seek to identify and optimize the best strategies for reducing the scale-up penalty and deliver the best possible impact on the outcome of interest—young children’s physical activity, health and development.

## Supplementary Material

daaf145_Supplementary_Data

## Data Availability

All data presented in this study are available in text and Supplementary material.
