# Supplementary material for: Scalability and scaling-up strategy of a physical activity policy intervention in Australian childcare centres
Source: Health Promot Int. 2025 Sep 3;40(5):daaf145. doi: 10.1093/heapro/daaf145 (PMC12404913; doi:10.1093/heapro/daaf145)
Supplement: daaf145_Supplementary_Data [file daaf145_supplementary_data.docx]

**Supplementary File 1**

**The Intervention Scalability Assessment Tool**

[**PART A: SETTING THE SCENE**](#_bookmark12)

Include background information on the public health problem, the context in which it is proposed the intervention will be scaled up and describe the intervention.

[**PART B: INTERVENTION IMPLEMENTATION PLANNING**](#_bookmark18)

Consider the implementation and feasibility factors relating to all potential scale up aspects, including fidelity and adaptations, reach and acceptability, delivery settings and agents, and implementation infrastructure and training.

**PART A: SETTING THE SCENE**

**Proposed level and method of scale up**

**Name of intervention: Play Active**

**The following questions set the scene for completing the tool. They relate to the level of scale up and the likely scale up pathway being considered.**

**Question 1: What is the ultimate level of scale up you are trying to achieve? (Select as many as necessary)**

- Scaled up across multiple sites within a region
- Scaled up across a local region or province

✔State or large jurisdiction scale up

- National or country level scale up
- Other level of scale up *(please describe)*
- Don’t know

**Question 2: How are you proposing to scale up?**

- Vertical approach (Simultaneous approach)

Note: Scaling up using a **vertical approach** involves the introduction of an intervention simultaneously across a whole system and results in institutionalisation of a change through policy, regulation, financing or health systems change.3

- Horizontal scale up (Stepwise approach)

Note: Scaling up using a **horizontal approach** involves the introduction of an intervention across different sites or groups in a phased manner, often beginning with a pilot program, followed by stepwise expansion, learning lessons along the way to help refine further expansion.3

- Other Vertical and Horizontal:

**Piloting a Vertical approach by making the Play Active program available to all Early Childhood Education and Care (ECEC) services in Western Australia, Queensland and South Australia; but also a Horizontal approach, refining the program based on lessons learnt.**

## DOMAIN A1: THE PROBLEM

This domain considers the problem being addressed. In this domain, describe the problem, who it affects, what it affects and how it is currently being addressed (if at all). Where possible, draw on recent data available that provides evidence of the problem and its impacts. This may include population survey data either at the local, regional or national level, or secondary data sources as examples.

| **Domain A1: The problem** | |
| --- | --- |
| **The problem – Describe what is known about the current nature of the problem** | |
| **A1.1: What is the problem and who does it affect?**  **Describe the nature and scope of the problem using epidemiological data (e.g. Who is affected? How widespread is the problem? What is known about the causes?)** | Low physical activity levels among children aged 0-5.  Our ‘PLAYCE Study - Childs Play’ report presents findings based on device-measured physical activity data for 1,596 children (aged 2-5 years) from 104 Early Childhood Education and Care (ECEC) services in Perth, Western Australia. Only 34% of 2-5 year old’s achieved the recommended 180 minutes of physical activity per average day at ECEC (1).Within Western Australia, there is no data on pre-school children’s physical activity levels at ECEC outside of metropolitan Perth, Western Australia.  In Queensland, (2) device-measured data found that less than half (47%) of children met both the 180-minute total physical activity guideline and 60 min of moderate-to-vigorous (energetic) intensity physical activity recommendations (2), when standardized to an 8-hour day.  In South Australia, we were not able to find any data for physical activity levels within childcare. However, levels are unlikely to be much higher than Queensland or South Australia. Overall in Australia, pooled device-measured data suggests that on an average 8-hour ECEC day, Australian preschool-aged children spend between 269 and 292 minutes, or approximately 60 percent of their day in movement (3). In contrast, Australian pre-school aged children only accumulate between 15 and 29 minutes of energetic play (i.e., moderate-to-vigorous intensity physical activity).  The major influences (causes) of physical inactivity in ECEC include the physical environment (e.g. space, equipment), as well as educator practices (e.g. routines and opportunity provision for play) (4).  ECEC is recognized as an important environment to support children’s physical activity (5, 6). |
| **A1.2: How does the problem impact on the health of the population?**  **Describe the impact of the problem on health and wellbeing of the population (e.g. burden of disease and costs to the health system and society)** | Physical inactivity contributes in this age group (5, 7) to:   - Poorer physical and mental health - Poorer sleep - Poorer motor skill development - Learning and behavioral issues - Higher levels of overweight and obesity   Physical (in)activity behavior tracks across childhood into adulthood (8). |
| **A1.3: What is current practice to address the issue?**  **Describe how this problem is currently addressed in the system** | Only 16% of ECEC services in Western Australia included children’s physical activity in their service policies (1). Similarly, few ECEC services in Queensland and South Australia include children’s physical activity in their service policies.  There is no current Western Australian, South Australian or Queensland legislation or regulation for ECEC services to have a physical activity policy.  Yet the Australian National Quality Standard 2.1 (6) state that: ‘Each child’s health and physical activity is supported and promoted’.  Including: Element 2.1.3: ‘Healthy eating and physical activity are promoted and appropriate for each child’. |

**Readiness assessment Domain A1: The problem**

| **Question** | **Not applicable** | **Not at all** | **To a small extent** | **Somewhat** | **To a large extent** |
| --- | --- | --- | --- | --- | --- |
| **1. Is the problem of sufficient concern to warrant scale up of the intervention/program to address it?** | **N/A** | **0** | **1** | **2** | **3** |
| **Total score for Domain A1** | ***Average scores included in main text*** | | | | |

## DOMAIN A2: THE INTERVENTION

This domain requires a description of the proposed program or intervention to address the problem described in A1. The aims, objectives, key elements and methods of delivery should be documented.

| **Domain A2: The program/ intervention** | |
| --- | --- |
| **Current situation – Describe what is currently known about the intervention proposed for scale up** | |
| **A2.1: Describe the aims/objectives and intended outcomes of the intervention proposed for scale up. Provide:**  **(1) a summary description of the intervention and**  **(2) its aims and objectives** | Play Active consists of an evidence-informed policy comprised of 25 practices and nine age-specific recommendations on the amount of physical activity, sedentary behaviour and screen time is recommended for children while in ECEC (9). To support services to implement the policy, there are 12 implementation support strategies for scale-up, adapted from the RCT (Mclaughlin et al, manuscript under review). The intended outcome is that the policy is uptaken and personalized by ECEC Directors, then implemented, which improves educators physical activity practices, in-turn increasing children’s energetic play at ECEC.  The primary aim of the Play Active scale-up trial will be to examine the implementation of Play Active in Western Australia, Queensland and South Australia. Specifically, the:   1. **Implementation** (primary aim): To assess the real-world effectiveness of Play Active’s scalable implementation support strategies for supporting ECEC services to adopt practices included in the Play Active policy. 2. **Effectiveness**: To assess if improvements in Play Active practice adoption (primary aim) are associated with improvements in educator-reported and device-measured (sub-sample) children’s physical activity. 3. **Sustainability**: Assess the effectiveness of the re-accreditation intervention on the sustainability of Play Activity policy practices. 4. **Dissemination**: Identify dissemination strategies which increase ECEC service registrations. 5. **Economic**: Conduct an economic evaluation to determine the cost-effectiveness of the intervention. 6. **Process evaluation**:   Objective A: To describe the re**ach, adoption and engagement** with Play Active in WA, QLD & SA.  Objective B: To describe the **acceptability, appropriateness, and feasibility** of the Play Active policy and implementation support strategies from the perspective of ECEC educators and directors.  Objective C: To describe **implementation barriers and facilitators** to Play Active. |
| **A2.2: Describe the key elements of the intervention proposed for scale up (including the process of delivery)**  **What are the key intervention components? (e.g. Frequency and intensity of the intervention, etc.)** | The policy (i.e., the intervention) contains 25 practices, including Manager, Supervisor and Educator practices (n=14); Physical Environment practices (n=4), Family and Carer practices (n=5) and Policy Monitoring and Review practices (n=2).  The 12 implementation support strategies, consisting of 48 sub-strategies are outlined in detail in Supplementary File 1. Briefly, they are:   1. Training (including state-specific online videos) 2. Tailoring of physical activity policy 3. Tailored prompts & positive reinforcement 4. Survey & feedback reports 5. Membership & accreditation 6. Resources (including state-specific resources) 7. Community of practice (state-specific) 8. Parent resources 9. Re-accreditation process 10. Branding & partnerships 11. Increase demand (recruitment/ dissemination strategy) 12. Co-development (study advisory & consumer group) |

**Readiness assessment Domain A2: Intervention characteristics**

| **Question** | **Not applicable** | **Not at all** | **To a small extent** | **Somewhat** | **To a large extent** |
| --- | --- | --- | --- | --- | --- |
| **2. Will the outcomes delivered by this intervention address the needs of the target group (and/or) problem?** | **N/A** | **0** | **1** | **2** | **3** |
| **Total score for Domain A2** | ***Average scores included in main text*** | | | | |

## DOMAIN A3: STRATEGIC AND POLITICAL CONTEXT

This domain requires consideration of the current strategic, political, and environmental context. It may also help to consider other influences that may contribute to the context such as industry/commercial players or the non- government sector.

| **Domain A3: Strategic and political context** | |
| --- | --- |
| **Strategic and political context – Describe what is known about the current strategic/political/environment context** | |
| **A3.1: Is addressing the problem consistent with national, state or regional policy directions or priorities?**  **(Yes, No, Don’t know). Provide evidence where possible to justify assessment.)** | Yes. Australian National Quality Standards 2.1 (6) state that:  Standard 2.1: ‘Each child’s health and physical activity is supported and promoted’.  Including: Element 2.1.3: ‘Healthy eating and physical activity are promoted and appropriate for each child’.   - Education and Care Services National Regulations 2012: Regulation 168 – Policies and procedures – 2 (a) Health and safety - Education and Care Services National Law (WA) Act 2012: Section 168 Offence relating to required programs - Early Years Learning Framework: Learning Outcome 1 – children feel safe, secure and supported; Learning Outcome 3 – children have a strong sense of wellbeing; Learning Outcome 4 – children are confident and involved learners; - Principles – secure, respectful, reciprocal relationships; respect for diversity; partnerships with families; ongoing learning and reflective practice - Practice – holistic approaches; intentional teaching; learning environments - There is currently no mandated need for a physical activity policy in Western Australia, South Australia or Queensland. Whereas there is a mandate for a policy on nutrition and sun safety. - Australian 24 Hour Movement Guidelines for the Early Years (7) |
| **A3.2: Is addressing the problem an identified need of funding agencies?**  **(Funding in agencies in this case may include central agencies such as NHMRC, Treasury, Cabinet Office, individual departments, non-government organisations or other advocacy groups)**  **(Yes, No, Don’t know). Consider if there are any**  **targets/indicators/goals at the local or international level that need to be met.** | Yes. Physical activity is a priority for the Western Australia government funding authority ‘Healthway’.  The federal government target in Australia is to reduce physical inactivity in the adult population by 15% by 2030, in line with the World Health Organization Global Action Plan on Physical Activity (2018-2030) (10).  Physical activity is also a priority for the Play Active Partner Advisory Group, led by the Telethon Kids Institute, which consists of:   1. Goodstart Early Learning 2. Early Childhood Australia 3. Australian Childcare Alliance, WA 4. Australian Childcare Alliance QLD 5. Australian Childcare Alliance SA 6. Play Matters Collective 7. Health and Wellbeing QLD 8. Cancer Council, WA 9. YMCA WA Child Care 10. Department of Local Government, Sport and Cultural Industries, WA   The following are supporters of Play Active:   1. Play Australia 2. Nature Play Australia 3. Department of Health, WA 4. Minderoo Foundation, CoLab for Kids 5. Sonas Early Learning 6. Sagewood Early Learning   We have also communicated with these stakeholders regarding Play Active:   1. National Heath Foundation 2. Australian Children’s Education and Care Quality Authority (ACECQA) 3. The Education and Care Regulatory Unit (ECRU) |
| **A3.3: How well will the intervention proposed for scale up align with the broader strategic and/or political context?**  **Consider if there are any political, strategic or environmental priorities or strategies this intervention might align with.**  **Also consider the influence of industry and private and non- government sector.** | The Play Active intervention aligns with the National Quality Standards 2.1 ‘Each child’s health and physical activity is supported and promoted’ (6), by providing guidance, resources and training on how to achieve this standard.  There is currently no mandate in Western Australia, Queensland or South Australia for ECEC services to have a physical activity policy, however, strategic advocacy is working towards this. In 2021, a submission was made to the National Quality Standards to “Amend the National Regulations to require services to have and implement a physical activity policy. Services will be required to develop new policies, or update existing policies, in order to meet Commonwealth Department of Health 24-Hour Movement Guidelines for the Early Years” (7). This however, was unsuccessful, with priority given to safety changes. |

**Readiness assessment Domain A3: Strategic/political context**

| **Question** | **Not applicable** | **Not at all** | **To a small extent** | **Somewhat** | **To a large extent** |
| --- | --- | --- | --- | --- | --- |
| **3. Is addressing the problem**  **consistent with policy/strategic directions or priorities?** | **N/A** | **0** | **1** | **2** | **3** |
| **4. Will scaling up the intervention be strategically useful to**  **funders/funding agency?** | **N/A** | **0** | **1** | **2** | **3** |
| **Total score for Domain A3** | ***Average scores included in main text*** | | | | |

## DOMAIN A4: EVIDENCE OF EFFECTIVENESS

This domain considers the level of evidence available to support the scale up of the proposed intervention. This includes the consideration of evidence from various sources such as the scientific literature and/or from results of any other known evaluations of the intervention if it has been piloted/trialled in your area or by someone else in another area. In some cases, you may have access to both types of evidence, but in others you may be limited to one only. It is important that you read and consider both if available.

In this section, the target population is defined as the group of people the intervention is intended for. In some cases, your target population can be very specific, for example, those with a certain health condition/risk factor. In others, the target population can be broad, for example all those within a specific geographical area. It is important that a target population is defined as it will have an impact on future monitoring and evaluation activities.

| **Domain A4: Evidence of effectiveness** | |
| --- | --- |
| **Level of evidence available** | |
| **A4.1 What is the strength of evidence of effectiveness for the intervention in addressing the problem described in Domain A1 and A2, based on literature?**  [**National Health and Medical Research Council evidence levels**](https://www.nhmrc.gov.au/_files_nhmrc/file/guidelines/developers/nhmrc_levels_grades_evidence_120423.pdf) **I Systematic review of level II evidence demonstrating**  **benefit**   1. **RCT or cluster RCT demonstrating benefit** 2. **A pseudo-randomised controlled trial demonstrating benefit**   **III Comparative study with no concurrent controls (non- randomised experimental trial, cohort study, case-control study, interrupted time series with a control) demonstrating benefit**   1. **Comparative study without concurrent controls (historical control study, two or more single arm study, interrupted time series analysis without a control group) demonstrating benefit** 2. **Case series with either post-test or pre-test/post-test outcomes** | Recent umbrella review of systematic review level evidence demonstrates the benefit of physical activity interventions generally in ECEC, including three meta-analyses. (11)  Play Active was evaluated as a pragmatic cluster randomised trial within 79 ECEC services in Perth, Western Australia (12). Analysis of the primary objectives after a 3-month policy implementation period found:   - There were no significant changes in the amount of physical activity or energetic play educators provided to children or in the proportion of educators providing the policy recommended ≥180 minutes of physical activity/day or ≥30 minutes of energetic play/day for intervention compared to wait-listed comparison services. - There was a significant increase in the uptake of director-reported policy practices (p=0.034), but no change in the uptake of the subset of high impact and low effort policy practices. - Intervention group educators reported high awareness of the Play Active policy recommendations (90%). - Play Active acceptability was high among educators (83%) and directors (78%). - Fidelity and reach were high for most implementation support strategies (>75%).   Implementation outcome data found increases in policy practice (procedure) uptake, however after 3 months of policy implementation support, this did not translate into changes in effectiveness (i.e., children’s physical activity levels at ECEC, as reported by educators). |
| **A4.2 What was the size of the intervention effect (if known)?**  **(Mean difference, relative risk, odds ratio, hazard ratio, sensitivity, specificity and statistical significance)**  **Note: It is important to know that intervention effects generally decline from controlled setting to implementation at scale in the real world.** | Meta-analyses effect sizes from umbrella review (11):  Finch et al (2016) (13) = SMD 0.44 [95% CI 0.12-0.76] child device-measured physical activity levels  Hnaitiuk et al (2019) (14) = 2.88 (95%  CI = 1.54, 4.23) minutes/day device-measured physical activity levels.  Engel et al (2018) (15) = standardised mean difference = 0.23 [0.11–0.36].  Play Active RCT outcomes (16) found that at over half of educators at baseline reported meeting both these policy recommendations for physical activity provision children (64.4% wait-listed comparison, 57.1% intervention). At follow-up, again over half of educators reported meeting both these policy recommendations for physical activity provision children (66.7% wait-listed comparison, 58.7% intervention). However, there were no significant group, time, or group-by-time effects for meeting physical activity policy recommendations or for the daily minutes of time allocated for total physical activity and/or energetic play. |
| **A4.3 Describe core intervention components (as described in Domain A2) that contribute to intervention effectiveness (if known)** | See domain A2 and Supplementary File 1 for a complete outline of the implementation support strategies.  It is unclear which of the implementation support strategies are the ‘core’ components. It is most likely they will act synergistically in the system approach to addressing inactivity. |
| **A4.4 Is the effect size of the intervention meaningful from a population health policy perspective?**  **Note: A statistically significant difference, though a good start, is not necessarily a difference of policy/clinical significance.**  **Intervention effects of policy/clinical significance are meaningful changes on an individual or group that, if scaled up, can make a substantial improvement to the outcome of interest.** | Every move counts when it comes to physical activity. It is a meaningful difference if we can increase young children’s physical activity by as little as a couple of minutes per day. Prior interventions in ECEC have shown this level of effectiveness (11). Establishing physical activity patterns early tracks through childhood into adulthood (8), so it is important to establish movement behaviours in line with the 24 hour movement guidelines for the early years (7).  Play Active data did not find any statistical difference in physical activity provided to children during the RCT (16), however, it did find an increase in director-reported practice uptake, which is on the behaviour change pathway to a positive change in young children’s physical activity. |
| **A4.5 Did the intervention have differential effects on the target population?**  **Note: Differences in effectiveness amongst target populations/settings.** | We have not (yet) examined differential effects for the Play Active trial between groups, e.g., low and high income areas. |
| **A4.6 Did the intervention have any known unintended consequences and/or adverse outcomes that were reported (in the literature or elsewhere)?**  **Note: Unintended consequences can be positive or negative.** | None recorded. |
| **A4.7 Is there evidence that the intervention has a relative advantage over existing interventions to address the same problem?** | No. There is no evidence to suggest Play Active is better or worse than any other ECEC based physical activity interventions (e.g., Munch & Move in NSW). There has been no studies to compare these interventions specifically.  However, we know from NSW, the uptake of the Munch n Move has been vast (17). Almost 90% of ECEC services have staff trained in Munch & Move, demonstrating the feasibility of large reach across different groups of similar programs. |
| **A4.8 Has the intervention been implemented at a:**   1. **Larger scale (either in literature or elsewhere)** 2. **Other delivery settings (from original intention)? If Yes to either, was it found to be effective? Describe the results** | No. Play Active has not been implemented at a larger scale or other delivery settings, beyond the original RCT (12). |

**Readiness assessment Domain A4: Evidence of effectiveness**

| **Question** | **Not applicable** | **Not at all** | **To a small**  **extent** | **Somewhat** | **To a large**  **extent** |
| --- | --- | --- | --- | --- | --- |
| **5. Is there compelling evidence (from the literature or elsewhere) to indicate that the intervention is effective in addressing**  **the problem in the target population?** | **N/A** | **0** | **1** | **2** | **3** |
| **Total score for Domain A4** | ***Average scores included in main text*** | | | | |

## DOMAIN A5: INTERVENTION COSTS AND BENEFITS

This domain considers the known costs of the intervention delivery as well as any quantifiable benefits. Economic evaluation is dependent on information on the costs and benefits of programs. Methods include cost effectiveness analysis, cost benefit analysis, cost utility analysis, etc.4 In some circumstances, intervention costs may not be well known, but it is preferable that some indication of costs be gathered so that more informed consideration of scalability can be made.

| **Domain A5: Intervention costs and benefits** | |
| --- | --- |
| **Level of evidence available** | |
| **A5.1 What were the intervention costs reported (if available)?**  **Consider costs associated with start-up (e.g. building infrastructure, conducting training), costs associated with ongoing delivery as well as cost per participant or cost per unit of outcome** | A formal economic evaluation has not been conducted of Play Active.  The delivery of implementation support strategies in the RCT (12) involved Research Assistant and student time (email and phone call support). The following personnel were involved in the delivery of the intervention, and it’s evaluation:   - 0.8 FTE Research Assistants (referred to as Project Officer) (implementation and evaluation) - 0.4 FTE Intern Students (contribute to implementation) - 0.8 FTE Senior Research Officer (evaluation and oversee implementation)   The program also involved providing free access to professional development courses (provided by KIDDO and Nature Play WA), that would normally incur a combined cost of AUD$935 per participant ($686+$249 for Nature Play and KIDDO, respectively). 255 educators were provided free access to this training, at an equivalent cost of AUD$238,425. |
| **A5.2 Was there any evidence of benefit outweighing the costs?**  **Describe any evidence that the benefits of the program outweighed the costs**  **Potential measures may include: incremental cost- effectiveness ratio, cost-benefit analysis, cost per QALY etc.** | No data available for this specific intervention. |

**Readiness assessment Domain A5: Intervention costs and benefits**

| **Question** | **Not applicable** | **Not at all** | **To a small extent** | **Somewhat** | **To a large extent** |
| --- | --- | --- | --- | --- | --- |
| **6. Is there evidence that the benefits of**  **the intervention exceeded the costs?** | **N/A** | **0** | **1** | **2** | **3** |
| **Total score for Domain A5** | ***Average scores included in main text*** | | | | |

# PART B: INTERVENTION IMPLEMENTATION PLANNING

Part B is about consideration for implementation of the intervention (as implemented currently or in the literature), as well as the proposed implementation for scale up. This section covers four domains:

1. Fidelity and adaptation
2. Reach and acceptability
3. Delivery settings and workforce
4. Implementation infrastructure.

As noted previously, while there may be some overlap with the information sought here with information required for future scale up implementation planning, completing this tool does not negate the need for a more detailed implementation scale up plan, if scale up is found to be warranted. The questions in Part B are designed to promote early thinking about potential implementation needs and strategies that would contribute to its potential for scalability.

## DOMAIN B1: FIDELITY AND ADAPTATION

This domain considers whether there are any proposed changes to the intervention required for scale up. For example, if the original intervention (described in Domain A2) required the delivery of 10 separate elements and only 8 elements are to be delivered in the scale up, record this. Any known impacts of these changes should also be noted.

| **Domain B1: Fidelity and adaption** | |
| --- | --- |
| **Considerations for scale up –What might change from the current situation if the intervention is scaled up?** | |
| **B1.1: Will there be any changes and/or adaptations made to the intervention from what was described in Domain A2 if the intervention is scaled up?**  **(Yes, No, Unsure).**  **If Yes, please indicate what those changes will be. Note: Adaptions to intervention components may have positive or negative impacts on intervention effectiveness** | Briefly, the adapted Play Active intervention content (the policy including 25 practices) will remain consistent with the original trial. As described previously, the policy (i.e. the intervention) was developed through a Delphi process (9) and using data from different state-based studies in ECEC. However, numerous adaptations were required to the implementation support strategies.  The policy (‘the intervention’) will be supported by an adapted implementation support strategy. The main adaptation will be to the delivery modes of implementation support strategies, including using digital delivery through a mobile-friendly website, automated emails and automated SMS.  The scale-up implementation strategy for WA, QLD and SA will consist of 12 strategies, including 48 sub-strategies – tailored to each state. |
| **B1.2: Are those changes and/or adaptations likely to have any impact on the intended outcomes of the intervention as described in Domain A2?**  **(Yes, No, Unsure). If Yes, please indicate what those changes/impacts will be** | A detailed paper has been submitted (Mclaughlin et al, manuscript under review) that outlines the specific adaptations made for scale-up to the implementation support strateiges. In line with best practice, these adaptations were outlined in detail according to the FRAME framework (18-20). Briefly, fifty-three adaptations to Play Active were identified. Most (68%) were made to the ‘content’ of the implementation strategies (including aspects of their delivery). In practice, this involved changing the delivery mode of implementation support strategies from phone call and email support, to website-based delivery. More than half (56%) of adaptations involved ‘adding elements’ for scale-up. Most adaptations were ‘fidelity consistent’ (95%). The main goals for adaptations were related to ‘increasing the acceptability, appropriateness, or feasibility’ (45%), ‘decreasing the costs’ (19%) and ‘increasing adoption of the evidence-based practice’ (19%). Adaptations were primarily small to medium in size with most proposed to have a positive (n=87%) or neutral (8%) effect on the effectiveness of the intervention, rather than negative (4%). |
| **B1.3: How will intervention fidelity be monitored and maintained?** | Completion of each of the implementation support strategies will be monitored through website analytics to assess fidelity to uptake of the implementation support. |

**Readiness assessment Domain B1: Fidelity and adaptation**

| **Question** | **Not applicable** | **Not at all** | **To a small extent** | **Somewhat** | **To a large extent** |
| --- | --- | --- | --- | --- | --- |
| **7. Will the core components of the scaled-**  **up intervention be consistent with what was previously shown to be effective?** | N/A | 0 | 1 | 2 | 3 |
| **8. If the core components of intervention are to be changed/adapted from its original form during scale up, will the impact of the changes/adaptations likely**  **be favourable?** | N/A | 0 | 1 | 2 | 3 |
| **9. Can program fidelity be monitored**  **and/or maintained if implemented at scale?** | N/A | 0 | 1 | 2 | 3 |
| **Total score for Domain B1** | ***Average scores included in main text*** | | | | |

## DOMAIN B2: REACH AND ACCEPTABILITY

This domain considers the reach and acceptability of the intervention for the target population.

| **Domain B2: Reach and acceptability** | |
| --- | --- |
| **Previous/current situation** |  |
| **B2.1 Describe the target population for the intervention**  **Describe who was targeted in the literature or in pilot program** | Long-day ECEC services located in Perth and Peel, Western Australia were the target population for the Play Active RCT. Ineligibility criteria included: long-day ECEC services catering exclusively for children requiring specialist care, mobile preschools, and Department of Education and Communities preschools; ECEC services already involved in alternative RCTs currently underway in Perth, Western Australia; and ECEC services that have had a significant change in management within the last three months or expect a change in management in the next three months.  Within each service, all full- and part-time educators were eligible to participate in the Play Active Trial. |
| **B2.2 How were the target population identified and recruited?**  **Describe how the target population(s) were identified and/or recruited in the literature or pilot program, e.g. what recruitment strategies were used** | ECEC services were recruited via an expression of interest form available on a study partner’s website (Cancer Council WA). A launch event was used to publicise the new Play Active program and inform services how to express their interest in receiving the physical activity policy template and implementation support strategies. ECEC services who expressed interest were contacted by the research team using a combination of modes (e.g., email, telephone) to invite them to take part in the pragmatic trial. ECEC providers with multiple services were contacted and all services invited to participate. |
| **B2.3 What was the level of participation and/or completion rate in the target population?**  **Describe the level of participation and/or rate of completion of the target population in the literature or pilot program** | At the time of recruitment there were 557 eligible ECEC services in Perth and Peel, WA. Seventy-nine services participated in the study, representing 13.7% of the individual ECEC services and 10.8% of service providers in the region.  Of the 79 services, 40 were randomized to the intervention group. All intervention services completed their policy adaptation and review process and were provided with the Resource Guide and access to professional development and training. In total 255 educators and 40 directors participated in the intervention group of the RCT study. |
| **B2.4 Was the intervention acceptable to the target population?**  **Was there any evidence (from literature or pilot program) to suggest that the intervention was acceptable to the intervention population?** | Play Active acceptability was high among educators (83%) and directors (78%). |
| **Considerations for scale up - Consider what might change from the current situation if the intervention is scaled up** | |
| **B2.5 Describe the target population for the intervention at scale** | Availability to all educators and Directors of long day ECEC services in WA (n=776), QLD (n=1,744) and SA (n=445), supporting up to 100,000 children in these states. |
| **B2.6 How will the intended target group be identified and recruited at scale?** | A recruitment strategy is currently under development in partnership with our Partner Advisory Group. Early Childhood Education and Care services will be identified through the Australian Children’s Education and Care Quality Authority (ACECQA) national registers. The proposed strategy includes:   - Social media targeted advertising - Direct mailout (recruitment flyer) – targeted advertising - Through large service provider head offices, including our partners (e.g. Goodstart Early Learning) - Facebook Page – Play Active - Videos – to advertise the program on social media - Launch event in each state |
| **B2.7 Have there been any projections/estimations developed for scale up, to consider:**   - **Likely level of participation and/or completion rates of the target population** - **Likely required timeframe required to achieve desired level of participation and/or reach?**   **If yes, what are they and how likely are they to be achieved within available resources?** | Four years of funding (mid 2023- mid 2027) has been secured from the 2021 Medical Research Future Fund (MRFF) Maternal Health and Healthy Lifestyles. With this funding, we are aiming for a minimum target recruitment of 25% (n=741) of services across WA, QLD and SA. |
| **B2.8 Are there any foreseeable facilitators and/or barriers for reaching the target populations as part of the scale up process?**  **Facilitators or barriers in this case can be in terms of process, persons, practices, policies, budget** | A Senior Research Officer has been appointed to manage the development of the scaled-up program and the research evaluation. At the time of writing, recruitment is underway to identify Project Officers in each state (WA, QLD and SA). |

**Readiness assessment Domain B2: Reach and acceptability**

| **Question** | **Not applicable** | **Not at all** | **To a small extent** | **Somewhat** | **To a large extent** |
| --- | --- | --- | --- | --- | --- |
| **10. Does the intervention have the potential to reach the intended target population**  **at scale?** | N/A | 0 | 1 | 2 | 3 |
| **11. Is the intervention likely to be acceptable to the target population?** | N/A | 0 | 1 | 2 | 3 |
| **Total score for Domain B2** | ***Average scores included in main text*** | | | | |

## DOMAIN B3: DELIVERY SETTING AND WORKFORCE

This domain considers the setting within which the intervention is delivered as well as the delivery workforce. In this domain, we refer to the delivery setting as the ‘setting’ in which the intervention is to be implemented, for example, schools, canteens, community, child care centres, hospitals. The delivery organisation, on the other hand, refers to the individual organisations that will implement the intervention. Delivery organisations may be newly created for the purpose of scaling up or they may already exist. Finally, the delivery workforce refers to those directly involved in delivering or administering the intervention to the target population.

| **Domain B3: Delivery setting and workforce** | |
| --- | --- |
| **Current situation** | |
| **B3.1 Describe the delivery setting/s where the intervention has been delivered**  **Describe where the intervention has been implemented (in pilot or literature). Delivery setting in this case means the context in which the intervention has been delivered e.g. schools, school canteens, sexual health clinics, community health centres, early childhood settings** | There are approximately 550 Long daycare ECEC services in Perth metropolitan region, Western Australia. 79 services were recruited, half were allocated to control. |
| **B3.2 Describe the delivery workforce required for the administering the program/ intervention**  **Who were they and what did they do?** | 0.8 FTE Senior Research Officer  0.8 FTE Research Assistants (referred to as Project Officer)  0.4 FTE Intern Students  The following were a series of tasks:   - Follow up on expression of interests to determine eligibility - Provided digital content via personalized emails (e.g. physical activity policy template, PDF resource guide) - Provide phone call prompts to personalize policy and select initial high impact low effort strategies to begin implementing - Reviewed policies and provided feedback on policies not meeting criteria - Sent out physical resources in mail (hard copy resource guide, accreditation certificate) - Liaised with training providers to provide user accounts and personalized emails - Phone call check-ins during policy implementation - Also involved in the trial evaluation (including sending online and paper surveys; face to face, phone call and email prompts for survey completion) |
| **B3.3 Was the intervention deemed acceptable to the delivery workforce?**  **For example, was there any feedback from the delivery workforce in relation to the intervention? Consider feedback from referrers to/from the intervention as well** | No data is available on the acceptability to the delivery workforce (i.e. the staff delivering the implementation support). Anecdotal evidence suggests the roles were acceptable. |
| **B3.4 How was the delivery workforce supported to deliver the intervention implementation?**  **For example, were resources provided to assist with**  **implementation, how much time was required to assist with implementation?** | The staff delivering the implementation support were trained and managed by the Senior Research Officer. |
| **B3.5 Were there any facilitators and/or barriers identified in the delivery setting when the intervention was implemented?**  **If yes, what were they?** | The early childhood sector has workforce barriers that may make it harder for services needing to incorporate new policy and practices. The United Workers Union (2021) identified the sector struggles with high staff turnover rates, financial stress, understaffing, excessive workloads, and additional stress due to COVID-19. News articles were also reporting additional staffing challenges due to COVID-19 vaccine mandates (21). |
| **Considerations for scale up – what might change from the current situation if the intervention is scaled up** | |
| **B3.6 Will the intervention be implemented in the same settings at scale?**  **Yes, No, Unsure. If no, in which other settings will the intervention be implemented?** | Very similar setting, except, not just metropolitan long day ECEC services but also regional and remote services too – across WA, QLD and SA. |
| **B3.7 Who will deliver the intervention at scale?**  **For example, will the same delivery workforce be used? Will the same referrers be involved at scale?** | The same delivery workforce will be used, however, more reliance will be placed on digital and automated delivery modes rather than Project Officer follow-up via phone call and email. Digital delivery modes will include a website, newsletters, Facebook group, automated email, and automated SMS. A single Project Officer will be co-located in each state, to deliver the intervention statewide in WA, QLD and SA, respectively, via the Play Active website. |
| **B3.8 Is the intervention likely to be acceptable to the delivery workforce involved if implemented at scale?** | Yes, more so now as it is shorter and easier to complete. This is mostly a result an automated policy review and approval process and shorter targeted online training. This directly combats some of the barriers raised by educators in the Play Active trial. |
| **B3.9 Does the intervention require a small or a large departure from current practices and cultures of delivery organisations and workforce?**  **Note: Consider the impact the implementation of the intervention will have on current practices and cultures of the organisation and whether those impacts will be well received** | A moderate departure from current practices, as there are environmental, scheduling, educational and educator physical activity-related practices to change. |
| **B3.10 Have there been any projections/estimations developed for scale up, to consider:**   - **Likely level of adoption/uptake rates of delivery organisations** - **Likely required timeframe required to achieve desired level of adoption/uptake by delivery organisations** - **Likely required timeframe to achieve the desired levels of resourcing/recruitment of the delivery workforce**   **If yes, what are they and how likely are they to be achieved within available resources?** | Play Active will be made available to long-day ECEC services statewide in WA, QLD and SA.  The target recruitment for the MRFF grant is 25% of ECEC services across WA, QLD and SA.  The likely timeframe required to achieve this desired level of uptake is within 6 months of launching the recruitment strategy.  The workforce is currently being recruited at the time of writing (December 2022). It is expected that the workforce will be in place for the launch of the recruitment strategy in April 2023. |
| **B3.11 Are there similar programs/interventions already in place in the proposed delivery setting that might facilitate or hinder scale up?**  **Does the intervention duplicate other services or interventions already in place or link with or leverage existing settings and/or services?** | There are similar programs for sun protection e.g. Cancer Council’s Generation SunSmart. As this is for another related-health behavior it is seen as complementary. Play Active has been modelled on this program which as a >90% uptake. In addition, the Cancer Council WA are partners of the Play Active project.  KIDDO is a similar program, except it is focused on train educators on children’s physical literacy. KIDDO are no longer a partner on Play Active. It is possible KIDDO could hinder the uptake of Play Active, if ECEC services get confused by the two programs and do not consider the importance to have and implement a service level physical activity policy. |
| **B3.12 Are there any foreseeable facilitators and/or barriers for the delivery settings as part of the scale up process?**  **Facilitators or barriers in this case can be in terms of process, people, practices, policies, budget** | A key facilitator is the funding in place from the MRFF. |

**Readiness assessment Domain B3: Delivery setting and workforce**

| **Question** | **Not applicable** | **Not at all** | **To a small**  **extent** | **Somewhat** | **To a large**  **extent** |
| --- | --- | --- | --- | --- | --- |
| **12. Is the delivery setting(s) selected to**  **deliver the program at scale consistent with that used in previous studies?** | N/A | 0 | 1 | 2 | 3 |
| **13. Is the delivery workforce selected to deliver the program at scale consistent**  **with that used in previous studies?** | N/A | 0 | 1 | 2 | 3 |
| **14. Is the intervention likely to be acceptable to the delivery workforce involved in its**  **delivery at scale?** | N/A | 0 | 1 | 2 | 3 |
| **15. If the intervention requires integration into existing organisational or community structures, how likely is it to**  **be feasible?** | N/A | 0 | 1 | 2 | 3 |
| **Total score for Domain B3** | ***Average scores included in main text*** | | | | |

## DOMAIN B4: IMPLEMENTATION INFRASTRUCTURE

This domain requires consideration of the potential implementation infrastructure required for scale up. Some of the answers to these questions may be known or could be extrapolated given known information.

For the purposes of the ISAT, implementation infrastructure comprises the organisational and workforce support systems required for implementation at scale, including training, accreditation processes, competency frameworks, information and performance monitoring systems.

Implementation support team for the purposes of the ISAT can be taken to refer to the additional human resources required to assist in the implementation at scale. Their roles may include, but are not limited to, assistance with

the delivery setting and workforce, managing or providing oversight of the scale up process, training and providing advice.

| **Domain B4: Implementation infrastructure** | |
| --- | --- |
| **Current situation** | |
| **B4.1 Describe the infrastructure requirements for the delivery of the program/ intervention**  **i.e. classrooms, clinic facilities, sporting fields, community centres, IT equipment, etc.** | Emails  Phone calls  Access code provided for free access to training  Physical resources stored as mail packs (e.g. Resource Guide)  Project Officer time |
| **B4.2 Describe the operational requirements for delivery of the intervention**  **i.e. training, education, monitoring and feedback systems, accreditation processes etc.** | Training provided via third party access code  Completion of policy tracked by Project Officer |
| **B4.3 Were there facilitators and/or barriers to the creation and maintenance of implementation infrastructure?** | Yes, it involved a high workload of time from the Project Officer, deemed not feasible for scale-up. |
| **Considerations for scale up – what might change from the current situation if the intervention is scaled up** | |
| **B4.4 Have there been any projections/estimations made for scale up, to consider:**   - **Likely implementation infrastructure required** - **Likely resources and timeframe required to build or procure the implementation infrastructure?**   **If yes, what are they and how likely are they to be achieved within available resources?** | Website  Physical resources stored as mail packs  Facebook Group  New shorter online training modules  New shorter policy tailoring process  The MRFF grant funding will cover four years of salary support for Project Officers in each state, as well as the development of all the implementation support strategies. This includes 36 months of intervention delivery. This is budgeted to be approximately AUD$1,727,513 (approx. AUD$431,878 per year). |
| **B4.5 Will implementation at scale require the creation of an implementation support team?**  **If yes, could they be created within proposed resources?** | In short, yes. However, the size of the team will be deliberately small to reduce the intervention costs and improve the cost-benefit ratio.  Website includes training videos, editable policy template, membership, further training, resources (physical and online), as well as automated tailored prompts to return to the website for those that drop out.  Facebook group managed by a part time Project Officer. |
| **B4.6 Are there any foreseeable facilitators and/or barriers to building implementation infrastructure as part of the scale up process?**  **Facilitators or barriers in this case can be in terms of acceptability to workforce, changes to practice, workload, etc.** | Potential drop out, due to lack of buy-in as accountability drops due to lack of face-to-face contact and Project Officer time. |

**Readiness assessment Domain B4: Implementation infrastructure**

| **Question** | **Not applicable** | **Not at all** | **To a small extent** | **Somewhat** | **To a large extent** |
| --- | --- | --- | --- | --- | --- |
| **16. Are the implementation infrastructure**  **requirements of the intervention/program feasible for scale up?** | N/A | 0 | 1 | 2 | 3 |
| **Total score for Domain B4** | ***Average scores included in main text*** | | | | |

## DOMAIN B5: SUSTAINABILITY

The purpose of this domain is to consider the longer-term outcomes of the scale up, and how, once scaled up, the intervention could become sustainable over the medium to longer term. Some of these questions will be difficult to answer or, in some cases, impossible.

However, they are listed to promote thinking and to facilitate planning, which may increase the likelihood of future success. It is worth noting that ‘sustainability’ is context dependent and it will be necessary to consider your context when determining what timeframe would be appropriate for the intervention to be considered sustainable or how best to define what sustainability means.

| **Domain B5: Sustainability of the intervention** | | |
| --- | --- | --- |
| **B5.1 What level of integration into existing service delivery settings or organisations will the intervention require if scaled up?**  **Also consider whether the level of integration is feasible or sustainable** | The intervention is the policy, which contains 25 practices to support physical activity through play and energetic play and meet ECEC specific recommended daily minutes of physical activity and sedentary behaviour. ECEC services have a number of polices as required by the National Childcare Regulations and the National Quality Standards. The Play Active policy will align with other service health promoting policies around healthy eating, sleep and sun protection. |  |
| **B5.2 If the intervention is implemented at scale, will it require a large commitment of funds (initial or ongoing)?**  **If yes to either or both, consider if an internal funding model such as co-payment schemes or sourcing from other agencies is possible, or whether it would be feasible to**  **implement a self-funding model to pay for parts or all of the intervention through co-payments from individuals or organisations** | Partly self-funded, as a fee for Certification ($100AUD) will be required. However, Certification will not be compulsory. The membership fee covers the cost of receiving the physical resources in the members pack. |  |
| **B5.3 Is the proposed delivery workforce required for implementation at scale sustainable (e.g. financially and/or in terms of supply)?**  **Is there an alternative delivery workforce that can deliver the implementation at scale, e.g. using fitness leaders to deliver exercise classes instead of physiotherapists** | Yes, the minimal workforce to maintain the wesbite, Facebook group and send out physical resources in the mail is required. The intervention could be delivered by one of the Play Active partners however the process and costs would be unlikely to change considerably. |  |

**Readiness assessment Domain B5: Sustainability**

| **Question** | **Not applicable** | **Not at all** | **To a small extent** | **Somewhat** | **To a large extent** |
| --- | --- | --- | --- | --- | --- |
| **17. Is the level of integration of the intervention into delivery settings required for implementation at scale**  **sustainable?** | N/A | 0 | 1 | 2 | 3 |
| **18. Is the level of resourcing required to implement the intervention at scale**  **sustainable?** | N/A | 0 | 1 | 2 | 3 |
| **19. Is the delivery workforce selected for**  **implementation at scale sustainable?** | N/A | 0 | 1 | 2 | 3 |
| **Total score for Domain B5** | ***Average scores included in main text*** | | | | |

# References

1. Christian, H., Rosenberg, M., Trost, S., Schipperijn, J., Maitland, C., Trapp, G., Lester, L., Boruff, B., Thornton, A., Zubrick, S., Powell, J. , Wenden, E. (2018). ‘A snapshot of the PLAYCE project: Findings from the Western Australian PLAY Spaces and Environments for Children’s Physical Activity Study. Supportive Childcare Environments for Physical Activity in the Early Years. Perth, Western Australia: The University of Western Australia, School of Population and Global Health. Accessed 28/02/2022. Available from: <https://www.telethonkids.org.au/globalassets/media/documents/brain--behaviour/child-physical-activity-health-and-development/christian_2018_childs-play_playce-study-report-2018.pdf>.

2. Loprinzi PD, Trost SG. Parental influences on physical activity behavior in preschool children. Preventive medicine. 2010;50(3):129-33.

3. Trost SG, Rosenberg M, Nathan A, Schipperijn J, Jones J, Yoong S, Wolfenden L, Christian H. Population-based reference values for total physical activity and energetic play while attending childcare. Paper presented at the International Society for Behavioral Nutrition and Physical Activity (ISBNPA) Annual Meeting June 8 -10, 2021.

4. Tonge KL, Jones RA, Okely AD. Correlates of children's objectively measured physical activity and sedentary behavior in early childhood education and care services: A systematic review. Preventive medicine. 2016;89:129-39.

5. Standards for healthy eating, physical activity, sedentary behaviour and sleep in early childhood education and care settings: a toolkit. Geneva: World Health Organization; 2021. Licence: CC BY-NC-SA 3.0 IGO. Available from: <https://www.who.int/publications-detail-redirect/9789240032255>.

6. Australian Childhood Education and Care Quality Authority (ACECQA). 2022. Quality Area 2 – Children’s health and safety. Accessed 21/02/2022. Available from: <https://www.acecqa.gov.au/nqf/national-quality-standard/quality-area-2-childrens-health-and-safety>.

7. Okely AD, Ghersi D, Hesketh KD, Santos R, Loughran SP, Cliff DP, et al. A collaborative approach to adopting/adapting guidelines - The Australian 24-Hour Movement Guidelines for the early years (Birth to 5 years): an integration of physical activity, sedentary behavior, and sleep. BMC Public Health. 2017;17(Suppl 5):869.

8. Telama R. Tracking of physical activity from childhood to adulthood: a review. Obesity facts. 2009;2(3):187-95.

9. Christian HE, Cross D, Rosenberg M, Schipperijn J, Shilton T, Trapp G, et al. Development of physical activity policy and implementation strategies for early childhood education and care settings using the Delphi process. International Journal of Behavioral Nutrition and Physical Activity. 2020;17(1):131.

10. World Health Organization. Global action plan on physical activity 2018-2030: more active people for a healthier world. Accessed 31/03/2021. Available from: <https://www.who.int/news-room/initiatives/gappa>. Geneva: World Health Organization; 2018.

11. Lum M, Wolfenden L, Jones J, Grady A, Christian H, Reilly K, et al. Interventions to Improve Child Physical Activity in the Early Childhood Education and Care Setting: An Umbrella Review. International Journal of Environmental Research and Public Health. 2022;19(4).

12. Nathan A, Adams E, Trost S, Cross D, Schipperijn J, McLaughlin M, et al. Evaluating the effectiveness of the Play Active policy intervention and implementation support in early childhood education and care: a pragmatic cluster randomised trial protocol. BMC Public Health. 2022;22(1):306.

13. Finch M, Jones J, Yoong S, Wiggers J, Wolfenden L. Effectiveness of centre-based childcare interventions in increasing child physical activity: a systematic review and meta-analysis for policymakers and practitioners. Obesity Reviews. 2016;17(5):412-28.

14. Hnatiuk JA, Brown HE, Downing KL, Hinkley T, Salmon J, Hesketh KD. Interventions to increase physical activity in children 0–5 years old: a systematic review, meta-analysis and realist synthesis. Obesity Reviews. 2019;20(1):75-87.

15. Engel AC, Broderick CR, van Doorn N, Hardy LL, Parmenter BJ. Exploring the Relationship Between Fundamental Motor Skill Interventions and Physical Activity Levels in Children: A Systematic Review and Meta-analysis. Sports Medicine. 2018;48(8):1845-57.

16. Adams E, Nathan A, Trost S, Schipperijn J, Shilton T, Trapp G, Maitland C, Thornton A, Mclaughlin M, George P, Wenden E, Christian H. Play Active physical activity policy intervention and implementation support in early childhood education and care: results from a pragmatic cluster randomised trial. 2023. International Journal of Behavioral Nutrition and Physical Activity (IJNBNPA). In Press. .

17. Green AM, Mihrshahi S, Innes-Hughes C, O'Hara BJ, McGill B, Rissel C. Implementation of an Early Childhood Healthy Eating and Physical Activity Program in New South Wales, Australia: Munch & Move. Frontiers in Public Health. 2020;8.

18. Stirman. FRAME Coding Manual. Accessed: 22/04/2021. Available from: <http://med.stanford.edu/fastlab/research/adaptation.html>. 2020.

19. Miller CJ, Barnett ML, Baumann AA, Gutner CA, Wiltsey-Stirman S. The FRAME-IS: a framework for documenting modifications to implementation strategies in healthcare. Implementation Science. 2021;16(1):36.

20. Wiltsey Stirman S, Baumann AA, Miller CJ. The FRAME: an expanded framework for reporting adaptations and modifications to evidence-based interventions. Implementation Science. 2019;14(1):58.

21. Branley, A. & Duffy, C. 2021. Childcare COVID vaccine mandates causing staff shortages and fee hikes.
